# Towards the geometric structure of small supported Au_9_ clusters on Si

**DOI:** 10.1038/s41598-018-30750-w

**Published:** 2018-08-17

**Authors:** D. Chekrygina, A. Rothkirch, I. Baev, F. Kielgast, P. Pandit, W. Wurth, M. Martins

**Affiliations:** 1 Universität Hamburg, Luruper Chaussee 149, 22761 Hamburg, Germany; 2 Deutsches Elektronen-Synchrotron (DESY), Notkestraße 85, 22607 Hamburg, Germany

## Abstract

Ultra-small clusters containing few atoms are of high interest in both fundamental research and applications due to their specific functional, magnetic or chemical properties which depend on size and composition. The experimental results of the morphology of the size-selected clusters, consisting of few atoms can be an ideal benchmark for sophisticated theoretical models. With this motivation we have investigated the geometrical structure of mass-selected Au_9_ clusters deposited on a silicon substrate prepared by soft-landing conditions. We present results obtained experimentally by Grazing-Incidence Small-Angle X-ray Scattering (GISAXS). Considering the ultra-small size of the clusters and small quantities of material on the surface, we combined advanced techniques which allowed us to investigate the surface structure of the sample. The resulting structural sizes are in concordance with cluster theory. Using a model-based approach, the advanced X-ray techniques allow for understanding how to resolve the possible cluster structure, identify optimal experimental conditions and obtain the probable morphological information which is challenging to be obtained otherwise.

## Introduction

Supported metal nanoclusters are of great interest due to their unique properties which differ from those of the bulk material owing to a large surface to volume ratio and the order of confinement. Also, increasing or decreasing the size of a cluster by one can significantly change their properties, demonstrating the importance of every atom. The surface nature can also greatly affect the structure and thus the functionality of the clusters. Physical^1–4^ and chemical^5–7^ properties can be tailored up to some extent by controlling their size and geometrical structure. Studies have shown cluster size effects on the catalytic activity^8–11^, plasmonic properties^12–15^ and magnetic properties^16^. For example, an addition of a single atom in such a cluster can increase the catalytic activity by up to one order of magnitude^5^; hence size-selection is required^6^ for fabrication of catalysts^17–19^. Also deposited Au size selected nanoclusters can be used as an effective catalyst in synthesis of nanostructures, such as ZnO nanowires^20^. Au nanoclusters have proven to be effective in environmental applications of sensing toxic chemicals^18^. In XMCD measurements on mass selected supported clusters, both spin and orbital moments depend on the quantity of atoms in the system^16^. Therefore, ultra-small metal clusters containing only a few atoms are very promising candidates. For many applications it is usually essential to allocate them on a substrate because it is complicated to use them in the gas phase.

Ultra-small clusters such as gold (Au) (n < 20) can be prepared by chemical synthesis^21,22^ or by sputtering deposition technique.^23,24^. One example of the former method is the preparation of the solution such as Au_9_(PPh_3_)_8_(NO_3_)_3_ where mass-selected Au_9_ clusters are stabilized by the phosphate ligand as described by Wen *et al*.^25^. Afterwards such clusters can be deposited on the substrate, dried in vacuum and investigated^26^. The chemical preparation of such clusters requires stabilization with ligands to prevent agglomerations during solution based deposition methods. This can change the configuration during preparation or in the process of removing ligands (e.g. calcination, heating). In the latter technique, nanostructures can be prepared by using ion (RF) source^27,28^ or magnetron sputter source^29,30^. In these methods, clusters are created via self-assembly after bombarding the metal target with ions. These techniques are highly controllable due to the tuneable deposition rate and the clusters produced are initially in their most natural conformation^16,31^. Furthermore, it is also possible to obtain ligand-free mass-selected clusters, for example by ionising them and selecting by their mass over charge ratio. However, the geometry of nanoclusters in each of the methods mentioned above can be affected by the surface potential besides the chemical preparation or the kinetic energy of the deposition process.

The clusters tend to diffuse and aggregate on the metal surface at ambient temperatures, because of their very small diameter (<1 nm), whereas on oxide surfaces the mobility and agglomeration is strongly suppressed for temperatures below 250 °C^32,33^. Thus, to avoid agglomeration the amount of the material on the surface is limited to the sub-monolayer range and furthermore a protective layer is applied in order to achieve well-separated clusters and to preserve them. It is extremely important to have a balance between the preferable small amount of the material on the surface, the resolution of the investigation method and the transparency of the capping layer in the experiment. This means that there is the requirement for the investigation method to be sufficiently sensitive to small amounts of material and to penetration depth. A strong dependency of physical properties on the morphology of the clusters depicts the need of complete information on their size, shape and the crystallographic structure. This understanding will then provide desired geometrical structures, which manifest novel physical properties for device applications^4,18^. In view of this, in the present work sub-monolayer thin films of ultra-small mass-selected Au clusters are investigated by Small- and Wide-Angle X-ray Scattering (GISAXS and GIWAXS) techniques using microfocused synchrotron radiation. In grazing incidence geometry the penetration depth is restricted to the surface layer which is extremely important for investigation of the nanostructure on the surface. GISAXS provides the physical properties averaged over the whole sample. It can give complete structural information such as size, shape and spatial correlations, whereas, in GIWAXS one can get information about the crystallinity and crystalline size as it has atomic probing length. In parallel X-ray fluorescence (XRF) measurements have been carried out. Combined analysis of these measurements will in turn provide a deeper inside to the geometrical structure.

Ultrasmall mass-selected Au clusters are modelled using density functional theory (DFT)^11,34–37^ to predict their possible conformations depending on charge and amount of atoms in a cluster. In case of Au_9_, calculations show that such clusters may form a planar quasi two-dimensional (2D) structure as well as a three-dimensional (3D) structure^11,17^. In one recent publication, Au_9_ occur to be in both conformations^22^ for clusters prepared by the chemical route and stabilized with ligands.

In this work, we present a study on the structural properties of supported ultrasmall mass-selected Au_9_ clusters and adatoms. First, a comparison of the adatomic deposition using soft-landing scheme and sputter deposition is given, followed by a morphological analysis of mass selected Au_9_ clusters. The variation of cluster size, composition and intra-cluster interaction is discussed and the geometrical structure of Au_9_ deposited on Si is presented. In addition, benefits of the protective capping layer are examined. This study seeks to establish control over size selected growth of ultra-small gold nanoclusters using soft landed techniques and their structural stability in ambient conditions. The main focus of our research is the understanding of the morphology of deposited Au_9_ clusters and the comparison with the shape predicted by theory.

## Materials and Methods

In order to compare the geometrical structure of Au_9_ clusters and monoatomically deposited samples we focused on three samples:

1. Adatomic sample – monoatomic deposited sample with 50% Au monolayer (ML), where an ion cluster source^27^ was used.
2. Au_9_ sample – sample with 10% ML of deposited clusters consisting of 9 Au atoms, where an ion cluster source^27^ was used.
3. Sputtered sample – monoatomic deposited sample with 50% Au monolayer (ML), where a radio-frequency source^38^ was used.

Sample preparation details for all mentioned samples are given below.

### Sample preparation

Au clusters have been deposited on boron-doped single crystal (100) silicon wafers (Si-Mat, Germany). This substrate has shown suitable for investigation of ultrasmall Au_n_ samples, since it helps to maintain their properties as clusters^32^. Substrates were cut into squares of 9 × 9 mm^2^ using a diamond saw, ultrasonically cleaned with acetone for 15 min and subsequently rinsed with isopropanol and deionized water and followed by Piranha acid treatment^39^. This reproducibly creates a clean and smooth native oxide layer on the silicon surface, which increases the adhesion of the material to substrate^40^. Si wafers used are single crystalline and the oxide layer is at least partially ordered (bare substrate investigation is described in the Supported information). The wafers were then rinsed with a large amount of deionized water and kept in a heater at 80 °C for 3 hours to dry. For two of the samples, Au markers were deposited on the left and right edges of the substrates by using a RF-sputter deposition^38^ and a 5 × 10 mm² silicon wafer piece as shadowing mask. The resulting markers have a width of 2 mm and a 10 nm thickness of Au. They were important for investigation since they help to localize the cluster position on the substrate. Both such substrates were used afterwards for mass-selected deposition of the Au_9_ clusters and adatoms. Au with an effective thickness (amount of material if it would be homogenously distributed on the sample surface) of 0.144 nm was deposited with the above mentioned method^38^ on the whole substrate for comparison by RF sputter deposition.

**Figure 1:**
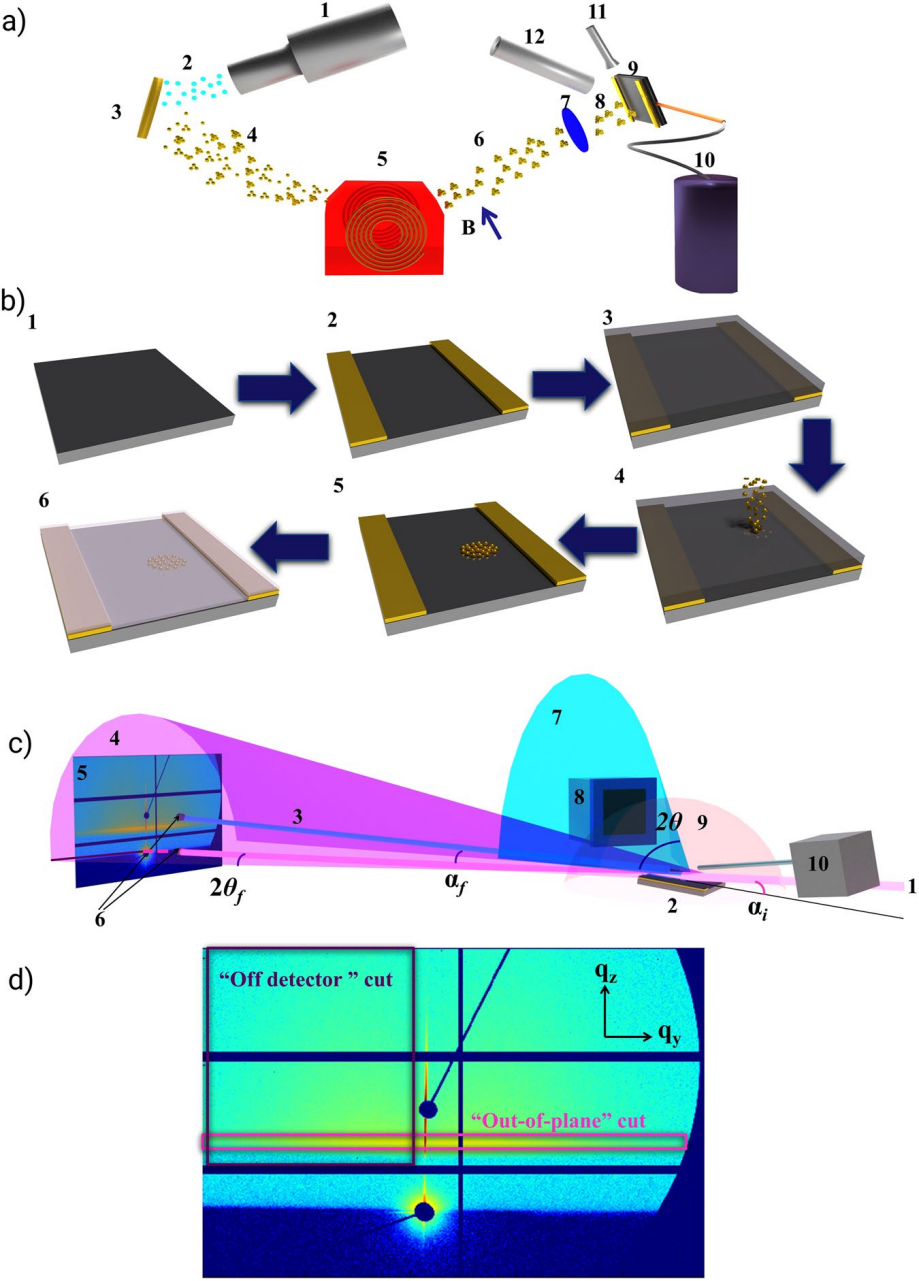
(**a**) Cluster sputtering source ICARUS. 1.Sputter gun. 2. Xe^+^ ions. 3. Au target. 4. Au clusters and adatoms. 5. Dipole magnet for mass selection. 6. Mass-selected clusters or adatoms. 7. Retardation lenses. 8. Mass selected clusters with E_kin_ <1 eV/atom. 9. Si substrate with Au markers. 10. He cooling for the substrate. 11. Kr gas shower. 12. Evaporator with Al. (**b**) Scheme of the sample preparation. 1. Si substrate 9 × 9 mm^2^. 2. Au markers (2 mm width, 10 nm height) sputtered on the edges. 3. Frozen Kr gas on the surface. 4. Soft landing of Au_9_ in Kr matrix in the spot with the radius of 1 mm. 5. Kr matrix removed and clusters landed. 6. Al capping layer evaporated on the system. (**c**) Scheme of the scattering experiment at P03. 1. Synchrotron X-rays. 2. Sample with Au clusters or adatoms. 3. Beam reflected from the sample. 4. Small angle intensity distribution. 5. Resulted GISAXS pattern on the detector Pilatus 1 M (Dectris). 6. Beamstops: primary (bottom), specular (top). 7. Wide angle intensity distribution. 8. GIWAXS detector Pilatus 300 K (Dectris). 9. X-ray fluorescence from the sample. 10. X-ray fluorescence spectrometer Vortex. Angles shown in the scheme: **α**_***i***_ – incident angle between the X-ray beam and the sample surface, which is <1°; **α**_***f***_ – exit angle in GISAXS; 2***θ***_***f***_ – out-of-plane angle in GISAXS; 2***θ*** – exit angle in GIWAXS. (**d**) Scheme of the typical cuts made in GISAXS experiments. In both cases the cuts with maximum q-range are shown.

Substrates with markers were placed in the deposition chamber in ultra-high vacuum conditions to prepare for the deposition of Au atoms (“Au_1_ clusters”) and Au_9_ clusters. These clusters were produced in the ICARUS source^27^ by Xe^+^ ion sputtering using a high purity Au target (99,999%, MaTecK GmbH). The scheme of the experiment is given in Fig. 1a. Xenon ions (Xe^+^) were accelerated by high voltage of 30 kV and the Au target was set to a voltage of 500 V. The base pressure during sample preparation was maintained at 1 × 10^−9^ mbar. Positively charged atoms and clusters ejected by the Xe bombardment were accelerated and collimated by an electrostatic lens system and then mass separated by a dipole magnet^16^. Because of the particular qualities of the cluster source used such a correlation existed: the smaller the amount of atoms in cluster the bigger the yield (for the adatomic deposition it is the highest)^16^. The following soft landing scheme^41,42^ was used for the cluster deposition: 1) cluster ions were decelerated to a kinetic energy less than 1 eV/atom; 2) a rare gas buffer of 5–10 monolayers Kr were frozen onto the He-cooled sample. The buffer has proven to be effective in suppressing cluster fragmentation^43^. The sample with Au_9_ clusters was produced with the low amount of atoms deposited which corresponds to 10% of a gold monolayer to minimize inter-cluster interactions and agglomeration, while the adatomic sample had coverage of 50% ML. Monoatomic deposition provides the biggest deposition rates and this allowed us to get a thicker layer in a relatively small amount of time, which was important given our intention to prepare the sample as a reference to determine the location of the spot with size-selected clusters from the marker position. With this larger amount of Au in comparison to the other sample having 10% ML, the X-ray fluorescence yield is higher and thus the detection of the spot is more precise. For the sample with 50% ML the probability to aggregate is statistically higher than for 10% ML. Thus atoms of the adatomic sample form clusters, while Au_9_ clusters having only 10% ML mostly remain the same size. In our case, “spot” means the area where clusters are deposited. After deposition in the Kr matrix, the clusters were brought into contact with the surface by heating the sample to ≈170 K, sublimating the Kr layer. Although prior to deposition, clusters were positively charged, on the surface they are neutral. The resulting cluster spot had a maximum radius of 1 mm. Before removing the sample from the ultra-high vacuum (UHV) preparation chamber, an Al capping layer of thickness of around 5 nm was evaporated in order to suppress surface diffusion, to prevent nanocluster aggregation and to avoid chemical reactions of the clusters (e.g. oxidation)^44^. The layer thickness was deduced from preliminary measurements of deposition rates using a quartz microbalance. The capping layer allows measuring such samples *ex situ* at ambient conditions using advanced scattering methods which is crucial for the investigation of these ultrasmall geometrical structures when they are deposited on a very small area. A scheme of the sample preparation is shown in Fig. 1b.

### Sample investigation

The main method of investigation was GISAXS, which is a powerful scattering technique using grazing incidence geometry^45,46^ allowing surface sensitivity. This technique is non-destructive, yields characteristics of geometrical structures with statistical relevance, and the probing depth can be varied with the incident angle (which is crucial for buried particles). Using GISAXS, it is possible to investigate nano and microstructures, depending on the sample to detector distance. Additionally, in combination with the complimentary GIWAXS method, one can investigate crystallographic lattice parameters of the same sample. A synchrotron radiation source is required for investigation of such small structures. This is caused by the relation that the scattering intensity is proportional to the square of the cross section of such a nanoobject. Besides, due to the grazing incidence geometry also reflection events need to be taken in to account in the data analysis using the Distorted Wave Born Approximation^46–48^. The resulting scattering pattern is shown exemplarily in Fig. 1d. For analysis, data reduction can be applied by integrating the intensity distribution from the 2D scattering pattern in the horizontal direction along q_y_ (“out-of-plane”) and in the vertical along q_z_ (“off-detector”) at a finite q_y_ (typical ranges covered are e.g. (0.1 < q_y_ < 2.2) nm^−1^) (so-called “cuts”). In Fig. 1d the position of these cuts are shown. If the former is made at the critical angle of the substrate (e.g. for Si it is at q_z_ = 0.7 nm^−1^), this so-called Yoneda cut gives the information on correlations in the lateral electron density distribution, mainly the average interparticle distance, the size and the shape of the nanostructures respectively in the near surface regime^12^. Similarly, the off-detector cut projecting the intensity in the q_z_ direction contains electron density correlations perpendicular to the surface, e.g. the average nanoparticle height or the thickness and roughness of a layer^12,46^. To analyse these cuts we combine them into contour plots to show the changes with time, distance or thickness.

Prepared samples with mass-selected clusters were investigated at MiNaXS beamline P03, PETRA III, DESY, Hamburg, Germany^49^ using the setup depicted in Fig. 1c, which allowed simultaneous GISAXS, GIWAXS and XRF experiments. The complexity of characterisation of ultrasmall mass-selected clusters demands the combination of all these methods. Owing to this arrangement, it is possible to detect the geometrical position of the clusters on the substrate together with the determination of the radii and interclusteral distances as well as the crystalline sizes. The sputtered sample was also investigated at P03 but only with GISAXS as a reference.

In our experiments on mass-selected clusters, incident photon energy of 13 keV was used while for the sputtered sample an energy of 11 keV was used. In both cases a beam size of 20 × 30 μm² (H × V) under an incident angle of 0.45°-0.5° was used. The distance of the sample to the detector (Pilatus 1 M, Dectris Ltd.) was SDD = 2368 ± 2 mm for all experiments. It was chosen to cover the desired q-range, where objects as small as our ultrasmall clusters are visible. During investigation of mass-selected clusters GIWAXS and XRF were used simultaneously with GISAXS. The GIWAXS detector (Pilatus 300 K, Dectris Ltd.) was mounted such that it covered an angular range of about 21° < 2θ < 35°. This enabled measurements of the (111) and (200) diffraction rings of polycrystalline Au. The XRF detector (Vortex®-EM, Hitachi Inc.) was used for the detection of the sub-monolayer cluster spot on the surface. It was calibrated by X-ray emission lines of foils made of Ag, Tb, Rb, Mo, Cu and Ba and covered an energy range from 0 keV to ~14.6 keV in 2048 channels. Lateral scanning was performed along a distance of 7 mm with a stepping of 0.1 mm for the monoatomic deposited sample with 50% ML to localize the relative position of the cluster spot to the Au markers. For the sample of Au_9_ with 10%ML material 4 mm of the scanning distance has been covered according to the preceding scanning experiment. Several repetitions (>50) were made to increase the signal to noise ratio. The exposure time for each step was not exceeding several seconds on each spot during scanning to avoid beam induced effects and to prevent detector saturation. Two beamstops made of Pb were used to block the primary and the specular beam, which is usually required since the direct beam intensity can saturate or even damage the detector. Patterns originating from same positions were summed up for further data evaluation and q_y_ and q_z_ cuts were derived for the structural analysis on the critical angle of Si. For the sputtered sample was used only one frame with exposure time of 10 s on one position.

Additionally to the XRF and GISAXS studies, one year after the GISAXS measurements, the Au_9_ sample with 10%ML was investigated using X-ray photoemission spectroscopy (XPS) at the P04 beamline, PETRA III, DESY, Hamburg. This was done to prove the efficiency of the capping layer to be partially an oxygen scavenger for our system. The experiment was performed using the ARGUS instrument^50^ at 1000 eV photon energy. The photon energy and electron analyser settings were tuned to ultimate performance. The beamline bandwidth is roughly 50 meV and the analyser resolution is better than 20 meV. The fitted Lorentzian linewidth γ of the Au 4f_7/2_ line was 0.42 eV. The spectra were calibrated against the Au 4f_7/2_ line of a clean Au (111) crystal.

### Simulations

For a detailed analysis, simulations with IsGISAXS were carried out^51^. This proved to be an efficient method to confirm the morphological results obtained from experiments^14^. In the program, the particle form factor is calculated using Distorted Wave Born Approximation^52^. Additional to this, several assumptions were made: our particles are encapsulated in the layer on the substrate, they can be presented as simple geometrical shapes, the inter-clusteral distance and radius are those calculated from the experimental data, the capping layer is Al and the height cannot be smaller than the covalent diameter of Au atom (0.288 nm).

According to the results from DFT calculations^53^ we expect 2D or 3D shapes. A hemispheroidal shape was chosen as an optimal shape for modelling because it can illustrate the differences in such structures, when the height to radius aspect ratio is varied. Furthermore, it is the geometrically closest to the possible shape predicted by DFT^53^. All the simulations are discussed in detail in the result section and supplementary information.

## Results and Discussion

Here we present the results of our investigation of ultrasmall mass-selected clusters for the sample with 50% ML deposited monoatomic Au, with 10% ML deposited Au_9_ clusters and the sputtered sample with 50% ML Au. For each of the mass-selected samples, summed GISAXS and XRF data are shown for the repetitive lateral scan along the surface. For the sputtered sample, GISAXS data are shown in comparison with the adatomic sample having equivalent nominal thickness.

### Characterisation of the adatomic deposited and sputtered samples

**Figure 2:**
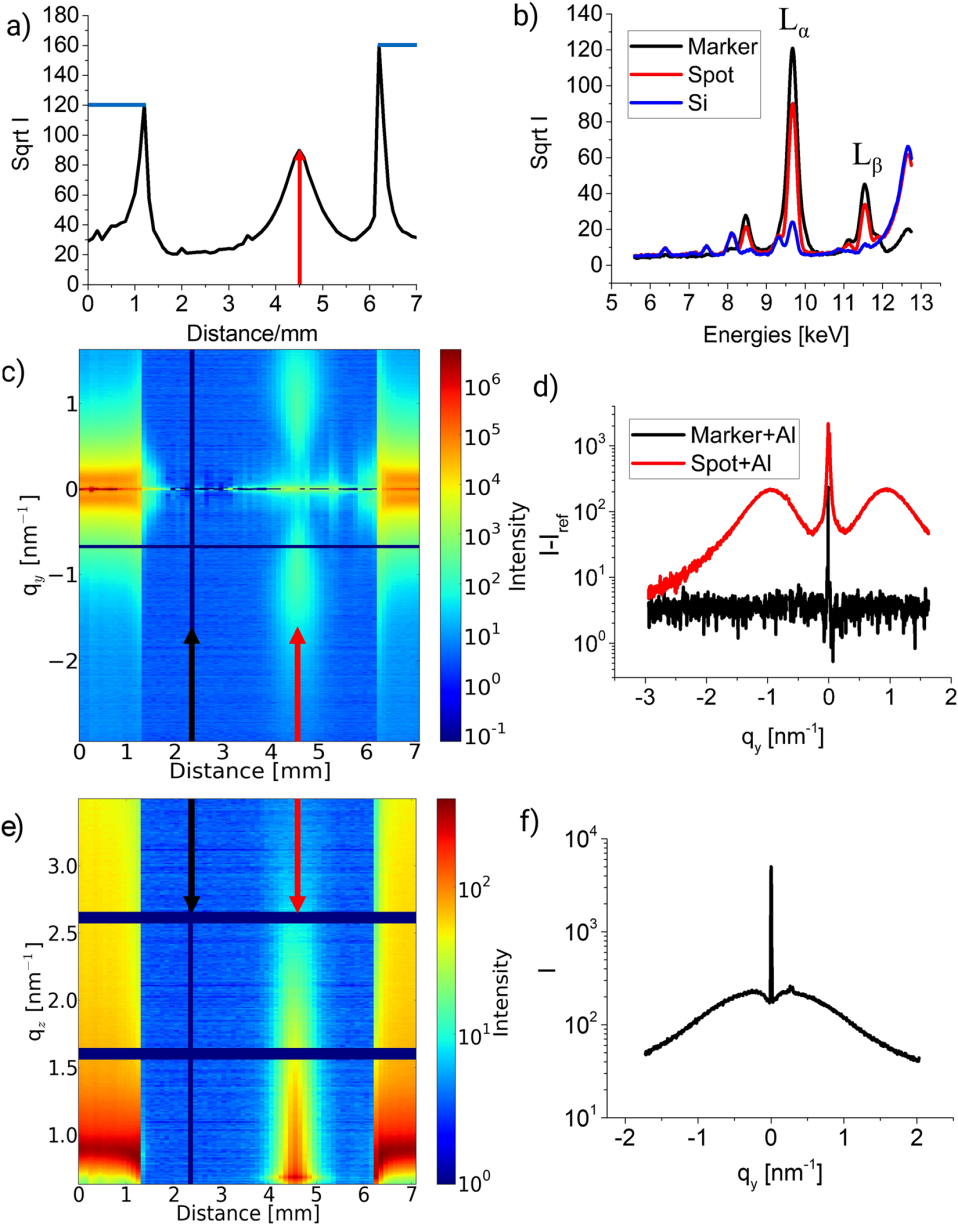
Fluorescence and scattering data for the sample with deposited 50% ML material of adatoms (**a–e**) and sputtered (**f**) clusters. All plots for the adatomic sample have Al capping layer on top, while sputtered is prepared without it. Scanned area of the former sample is 7 mm of the 9 mm of the sample surface. (**a**) XRF spectra of the sample at the L_α_ Au line as function of lateral distance on the Si surface. At a distance of 4.5 mm on x axis is the maximum of XRF signal stemming of the adatomic spot, on the left and right from it are Au markers. Blue lines show the expected shape of the markers, because the sharp shape of them caused by detector saturation. (**b**) XRF spectra of the sample for adatomic spot (red), Au markers (black) and Si (blue). (**c**) Contour plot of the out-of-plane cuts (along q_y_) derived at the Si Yoneda peak (q_z_ = 0.7 nm^−1^) for different positions on the substrate. The black arrow indicates the subtracted data frame, the red one-the maximum of the adatomic spot. (**d**) GISAXS out-of-plane (along q_y_) line cuts at the position with the maximum amount of material obtained from the XRF data. Red curve- adatomic spot, black curve- markers. **(e**) GISAXS off-detector (along q_z_) line cuts at q_y_ from −1.9 to −0.7 versus the position on the substrate. The black arrow indicates the subtracted data frame, the red one-the maximum of the adatomic spot. (**f**) GISAXS out-of-plane (along q_y_) line cuts for sputtered Au sample.

Figure 2a,b show the XRF plots for the sample of Au adatoms deposited in the soft-landing scheme with coverage of 50% of a ML. During the measurements the sample was moved perpendicular to the incident beam with small steps of 0.1 mm covering a range of 7 mm in total. This allowed the detection of part of the two Au markers at the edges and the Al-covered spot with ultrasmall Au clusters and thus it was possible to precisely locate the deposited cluster on the surface. Figure 2a gives the XRF yield at an energy of 9.6 keV (corresponding to the L_α_ – line of Au) as function of sample position. Given the scanning scheme and sample preparation, the left and the right peak correspond to the markers. The peak at position 4.5 mm (indicated with the red arrow) can only originate from the adatomic spot, also supported due to its width of about 1 mm (FWHM). Note that only part of the marker signals is seen and blue lines show the expected marker shape. In order to resolve the centre position of the low fluorescence at the cluster spot, the XRF detector at the markers shows saturation. Figure 2b shows the XRF yield measured at three selected positions on the adatomic sample, namely Au markers, substrate (Si) and spot. The emission lines of Au L_α_ and L_β_ are located at 9.6 keV and 11.4 keV, respectively, and are clearly visible in two of the spectra shown. Along with Fig. 2a, (see comments below), the spectra could be associated to the Au marker (red line), adatomic spot (black line) and the Al-capped Si substrate (blue line). The GISAXS contour plots given in Fig. 2c,e show how line cuts along q_y_ and q_z_ accordingly repeat the shape of the markers and the spot from Fig. 2a. Markers of Au, which show high signal intensity on the left and the right of the sample, are also recognizable. For all of the GISAXS plots Si and Al background was subtracted to enhance the Au signal. The black lines on the position 2.5 mm is from the subtraction of the Si background from the whole set of data. The Si and Al background was also subtracted from Fig. 2d to get a more prominent signal. Figure 2d depicts an out-of-plane cut from the location of the spot on the sample, whose position has been selected from the XRF signal. This graph gives information on the average lateral correlation distances of the objects. The peak position of the spot was used to estimate cluster correlation distances D by the formula^46^:

1 $${\rm{D}}=\frac{2{\rm{\pi }}}{{{\rm{q}}}_{{\rm{y}},{\rm{\max }}}\,}.$$

Given the position of the out-of-plane maximum at q_y,max_ = 0.91 nm^−1^ obtained by a Lorentzian fit of the intensity distribution in Fig. 2d, the average interparticle distance is about D = 6.9 nm. Further analysis was performed using the established hemispherical model for Au cluster growth on silicon substrates by Schwartzkopf *et al*.^14^. This model assumes the Au clusters to be locally organized as monodisperse hemispheres in a hexagonal arrangement^54^. In the present work we have calculated cluster radius. According to this model assumption, the average radius of the supported clusters can be calculated by^14^:

2 $$R=\sqrt[3]{\frac{{(\sqrt{3})}^{3}\delta {D}^{2}}{4\pi }},$$

where R is the radius of the cluster, δ is an effective thickness of a homogenous layer and D is the cluster correlation distance.

The results obtained are listed in Table 1.

**Table 1:** Calculated structural values for soft-landed adatomic and sputtered sample.

Name	Adatomic 50% ML	Sputtered 50% ML
Effective thickness δ [nm]	0.14 ± 0.01	0.14 ± 0.01
q_y,max_ [nm^−1^]	0.96 ± 0.01	0.29 ± 0.01
Cluster correlation distance D [nm]	6.54 ± 0.06	22.08 ± 0.59
Cluster radius R [nm]	1.37 ± 0.05	3.07 ± 0.02

Figure 2f depicts a typical out-of plane cut from the sputtered sample. Positions of the side maxima are located at smaller q_y_ than for the adatomic sample with equivalent coverage. This shows the existence of twice as large geometrical structures as for the sputtered sample. Since in the sputtered case small cluster sizes are expected^14^, this can indicate that the lack of a capping layer led to agglomeration. In the work of Levine *et al*., a cluster mobility for small Au amounts was detected^55^ which also can affect the adatomic sample. Although, our preparation method using soft-landing of ions and capping layer helps to make the agglomeration and cluster mobility effects lower. Therefore, not only the soft-landing scheme but a capping layer is mandatory in case of *ex situ* investigations of ultrasmall clusters.

### Characterisation of the Au_9_ deposited sample

**Figure 3:**
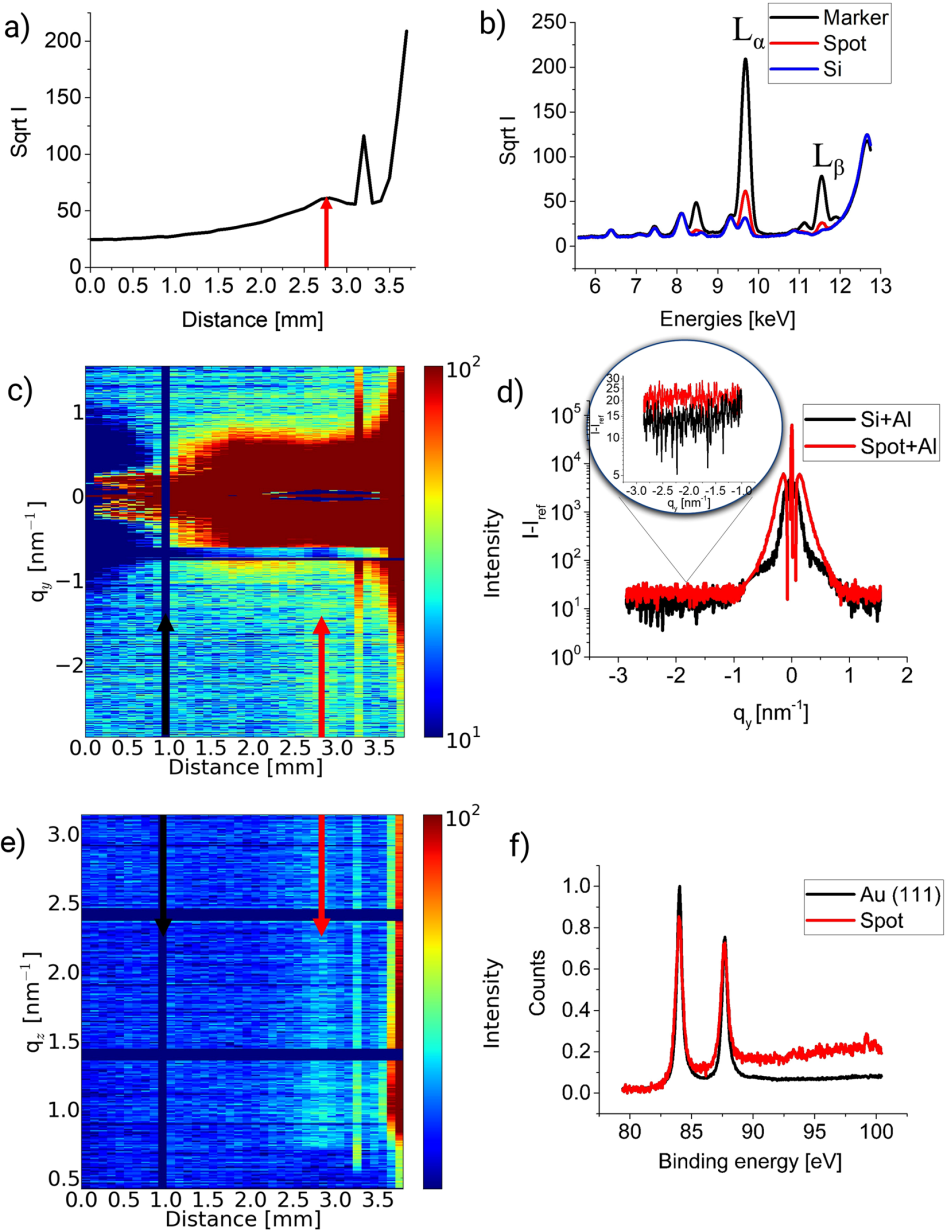
XRF, XPS and scattering data for the sample with deposited 10% ML material of Au_9_ clusters. All plots have Al capping layer on top. Scanned area of the former sample is 3.8 mm of the 9 mm sample surface. (**a**) XRF spectra of the sample at the L_α_ Au line as function of lateral distance on the Si surface. At a distance of 2.8 mm on x axis is the maximum of XRF signal stemming of the adatomic spot, on the right from it the sharp peak is an artefact and at 3.5 mm is an Au marker. (**b**) XRF spectra of the sample from energies for Au_9_ spot (red), Au marker (black) and Si (blue). (**c**) Contour plot of the out-of-plane cuts (along q_y_) derived at the Si Yoneda peak (q_z_ = 0.7 nm^−1^) for different positions on the substrate. The black arrow indicates the substracted data frame, the red one-the maximum of the Au_9_ spot. (**d**) GISAXS out-of-plane (along q_y_) line cuts at the position with the maximum amount of material obtained from the XRF data and the Si position. Red curve- Au_9_ spot, black curve- Si. The inset shows the difference between the Si and Au signal. (**e**) Contour plot of off-detector (along q_z_) line cuts at q_y_ from −2.1 to −1.4 versus the position on the substrate. The black arrow indicates the subtracted data frame, the red one the maximum of the Au_9_ spot. (**f**) XPS spectra for Au_9_ spot and Au (111) crystal at Au 4f_7/2_ line.

Figure 3 shows the corresponding set of plots as above for the Au_9_ soft-landing deposition. In this case we are using the same strategy but for much more complicated system with a 5 times lower deposited amount of Au, i.e. having a 10% ML coverage. This small amount of material is needed to reduce the probability of aggregation, thus allowing us to obtain the shape of the individual clusters. Figure 3a gives the fluorescent signal at E = 9.68 keV (L_α_ Au line) of the sample while scanning through it over a distance of 4 mm. Figure 3b shows the XRF yield collected on the Au_9_ sample for the Au marker (black), the Au_9_ cluster spot (red) and Si (blue). Figure 3c,e are illustrating contour plots obtained from corresponding GISAXS data. Both of these 2D GISAXS plots had an Al and Si background subtracted. To make it possible to see even a very weak signal from the size-selected cluster spot we adapted them. In Fig. 3a,c and e, a strong peak exists at distance of 3.2 mm from the start of the scan, which we consider being from the marker. In Fig. 3d out-of-plane cuts are shown for the cluster spot and Si. The inset shows a selected region ranging from −1 nm^−1^ to −3 nm^−1^ and suggests that a slight difference exists between the signals due to a peak occurrence in the spot signal at q_y,max_ = (2.4 ± 0.51) nm^−1^. However, the peak is very weak and may be assigned to a very small amount of the material on the surface, being consistent with the 10% ML coverage. For this sample the same hemispherical model^14^ as above was applied. The derived values for the cluster radii and correlation distances are summarized in Table 2. The radius for Au_9_ cluster results to 0.43 nm, which is close to the value expected for a 3D cluster where the base consists of 5 atoms, 3 on the 2^nd^ level and one on the 3^rd^. Such a cluster shape is one of those predicted by DFT calculations^11,53^. In view of possible changes induced by radiation (radiation damage), measurements were repeated with shorter exposure and didn’t show discrepancies with the existing data.

**Table 2:** Calculated structural values for soft-landed Au_9_ sample assuming hemispherical model.

Name	Hemispherical model
Effective thickness δ [nm]	0.03 ± 0.003
q_y,max_ [nm^−1^]	2.40 ± 0.51
Cluster correlation distance D [nm]	2.62 ± 0.56
Cluster radius R [nm]	0.43 ± 0.09

The XPS spectrum recorded is shown in Fig. 3f. It can be seen that the binding energy of the Au clusters agrees very well with the measured data from the Au (111) crystal. Also the binding energy of the Au_9_ clusters agrees very well with a comparable, previous experiment by Lim *et al*.^56^. Lim *et al*. have further shown that Au_9_ clusters can be oxidized in an activated oxygen atmosphere resulting in a large chemical shift of the Au 4 f lines, showing even more reactivity than smaller clusters^32^. Our results therefore imply that the Au clusters remain unoxidized due to the capping layer even after one year of storage under ambient conditions and after several hard X-ray measurements. Therefore possible effects such as beam damage or alloying with the capping layer can be ruled out.

No diffraction signal (GIWAXS) has been encountered for ultrasmall clusters. This was expected because of the low amount of material on the surface.

**Figure 4:**
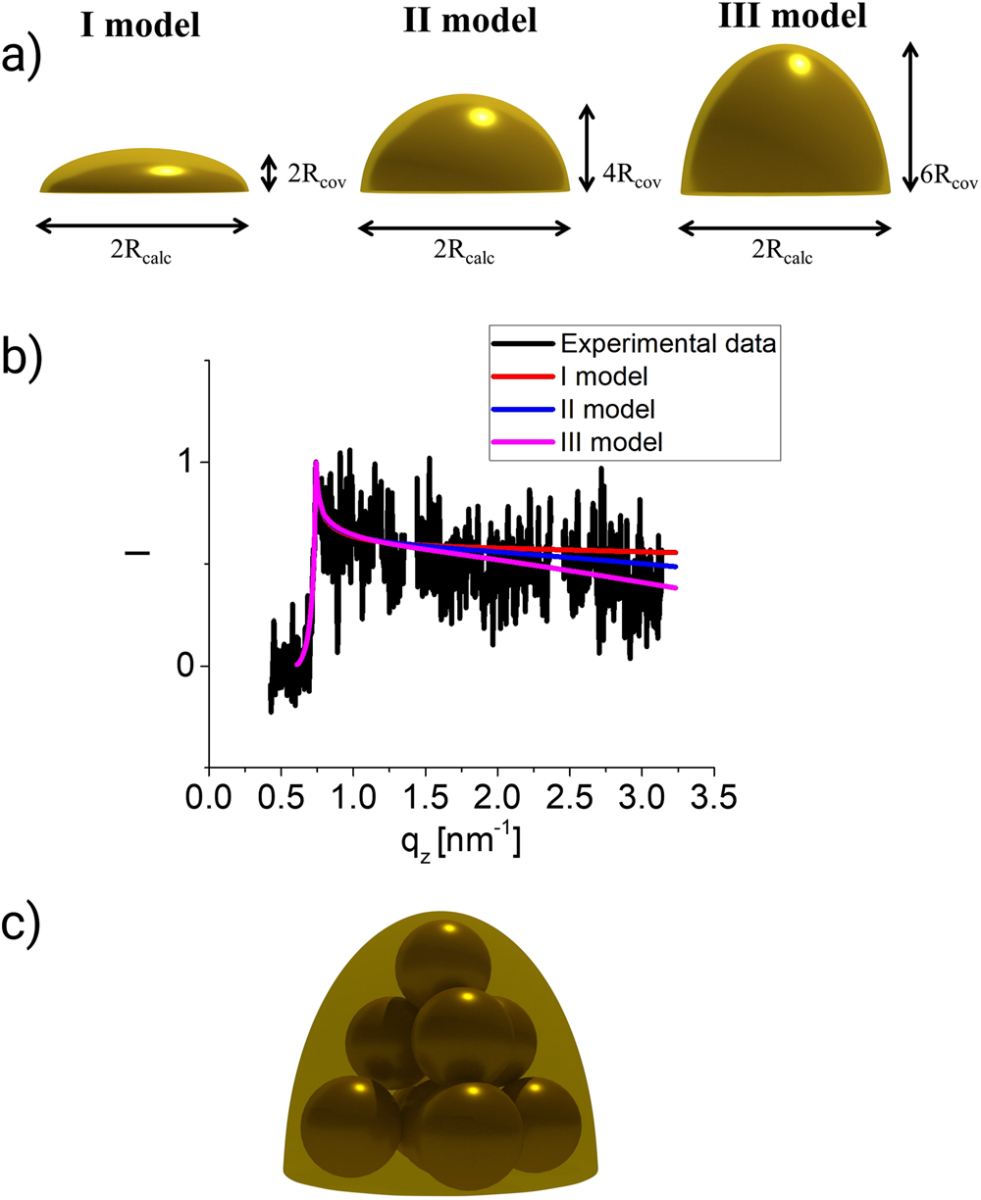
(**a**) Sketch of the hemispheroidal models as used in the IsGISAXS simulations. R_cov_ = covalent radius of Au. R_calc_ is the calculated radius for Au_9_ clusters. (**b**) GISAXS off-detector (along q_z_) line cuts for the simulated shapes and experimental data at the position with the maximum amount of material obtained from the XRF data. Pink curve - Au_9_ spot, black curve - hemispheroids with the height of 1 Au atom, red curve- hemispheroids with the height of 2 Au atoms, blue - hemispheroids with the height of 3 Au atoms. (**c**) Possible structure of the soft-landed Au_9_ on the Si surface according to the experimental data.

Figure 4a. shows the shapes used for simulating the possible geometric structure of our system. Hemispheroids were chosen since one can change the height without interruption of the radius. Constraints we set for these models: radius is constant and equal to that calculated from eq. 3; height cannot be smaller than the size of the Au atom; the size of the detector, wavelength and the sample to the detector distance is the same as in the real experiment; substrate is Si and Au clusters are covered with 5 nm of Al. Off-detector line cuts obtained from the simulated scattering patterns are compared with the experimental data in the Fig. 4b. These cuts contain information on the height of the object in the system. A linear fitting was done on the area from 1 nm^−1^ to 3 nm^−1^ for these curves to compare the slope between the simulations and the experiment because this can give a hint on the height of the cluster Au_9_. The values obtained are presented in Table 3. According to this the most probable structure has five atoms in the base, three on the 2^nd^ level and one on the 3^rd^. However, there can be clusters with differing shapes and sizes but due to the fact that GISAXS gives the average characteristics of the sample, the 3D one with the height of 3 Au atoms and the radius equal to 0.46 nm contributes the most. This is in concordance with one of the possible structures of Au_9_ clusters suggested by Fernandez *et al*.^53^ from DFT calculations and found experimentally by Schooss *et al*.^28^. Both of them made these models for clusters in the gas phase which indicates that the soft-landing deposition scheme preserved the 3D geometrical structure of these clusters. A sketch of the Au_9_ cluster obtained from our experimental data is shown in Fig. 4c.

**Table 3:** Slopes estimated for the linear fits of simulated data and experiment.

Name	Slope
I model	−0.0289 ± 0.0005
II model	−0.0620 ± 0.0004
III model	−0.1106 ± 0.0003
Experimental data	−0.094 ± 0.01

## Summary

In this study, we present an approach to obtain the geometrical shape for deposited ultrasmall metallic clusters using Au_9_ on Si/SiO_2_ as a case study. This was done using the GISAXS experimental technique which provided us with the average characteristics of the sample. For the first time we were able to obtain structural values using a scattering method on supported, small mass selected clusters indicating a preferential 3D shape of the Au_9_ clusters on the Si/SiO_2_ surface depicted in Fig. 4c with a radius R_Au9_ ≈ 0.43 nm. The proposed structure is similar to the 3D structure for Au_9_^+^ clusters in a gas phase.

To support this model, the data is compared to two different adatom samples. The adatom samples have been prepared using soft-landing and sputtering. The soft-landed adatom samples has also been capped with Al equivalent to the Au_9_ sample. The sputtered sample shows rather large nanoparticles with a mean radius R ≈ 3 nm, which can be explained by the enhanced mobility during the sputter preparation at room temperature and the lack of a capping layer. The mean size of the clusters of the soft-landed adatom sample is roughly two times smaller compared to the sputtered sample. In the WAXS data they also do not show a significant Bragg peak and only a weak and very broad signal. Hence, the soft-landed adatom structures have a rather stochastic geometric distribution as they are frozen on the cold sample during soft-landing and fixed by the capping layer at room temperature. The necessity of the capping layer also follows from the XPES investigation of the Au_9_ sample, where it was exposed to the ambient conditions for one year. The sample did not display the chemical shift expected for oxidized Au_9_ clusters. Comparing the radii of the soft-landed Au_9_ and adatom deposition the two different adatomic samples for the adatom samples 3x and 7x larger radii are found for the soft-landed and sputtered samples, respectively. This can be partially explained by the 5x larger amount of Au on the surface but also supports the proposed 3D structure of the Au_9_ clusters on the surface.

To summarize, we have described the challenge in the preparation of supported mass selected ultra-small Au clusters using a soft-landing technique. The results proved that *ex-situ* x-ray scattering and XPS measurements are possible after applying a capping layer protecting the samples. We introduced an experimental setup and an analysis approach which can be used to investigate low mass selected atomic sized geometrical structures. To our knowledge, this is the first probing of the geometrical structure of supported mass selected ultra-small cluster using GISAXS.

## Electronic supplementary material

Supplementary material
