# Supplementary material for: Towards the geometric structure of small supported Au9 clusters on Si
Source: Sci Rep. 2018 Aug 17;8:12371. doi: 10.1038/s41598-018-30750-w (PMC6098063; doi:10.1038/s41598-018-30750-w)
Supplement: Supplementary file 1 — Supplementary material [file 41598_2018_30750_MOESM1_ESM.docx]

**SUPPORTING INFORMATION**

Towards the geometric structure of small supported Au_9_ clusters on Si.

D. Chekrygina^1*^, A. Rothkirch^2^, I. Baev^1^, F. Kielgast^1^, P. Pandit^2^, W. Wurth^1, 2^ and M. Martins^1*^

^1^ Universität Hamburg, Luruper Chaussee 149, 22761 Hamburg, Germany

^2^ Deutsches Elektronen-Synchrotron (DESY), Notkestraße 85, 22607 Hamburg, Germany

*Corresponding authors: deniza.chekrygina@desy.de

michael.martins@desy.de

Comparison of soft-landing deposition of Au_9_ and non, using ion sputtering source ICARUS^1^*.*

Sample preparation:

Prepared samples:

Sample a: 10% ML of Au_9_, not soft-landed

Sample b: 3% ML of Au_9_, soft-landed

Pre-treatment of the Si substrate is the same as described in Material and methods of the manuscript. Setup used for the deposition is as in Fig.1a (main text). On both samples mass-selected Au_9_ clusters were deposited and then capped with Al. The main difference is that in the case of Sample a the Kr matrix was not prepared before the deposition. Also, it was cooled only till 86K.

Sample investigation:

Both samples were investigated at MiNaXS beamline P03, PETRA III, DESY, Hamburg, Germany. Photon energy used was 13 keV. The XRF detector (Vortex®-EM, Hitachi Inc.) was used for the detection of the Au cluster spot. It was calibrated the same as in sec. Materials and methods of the manuscript.


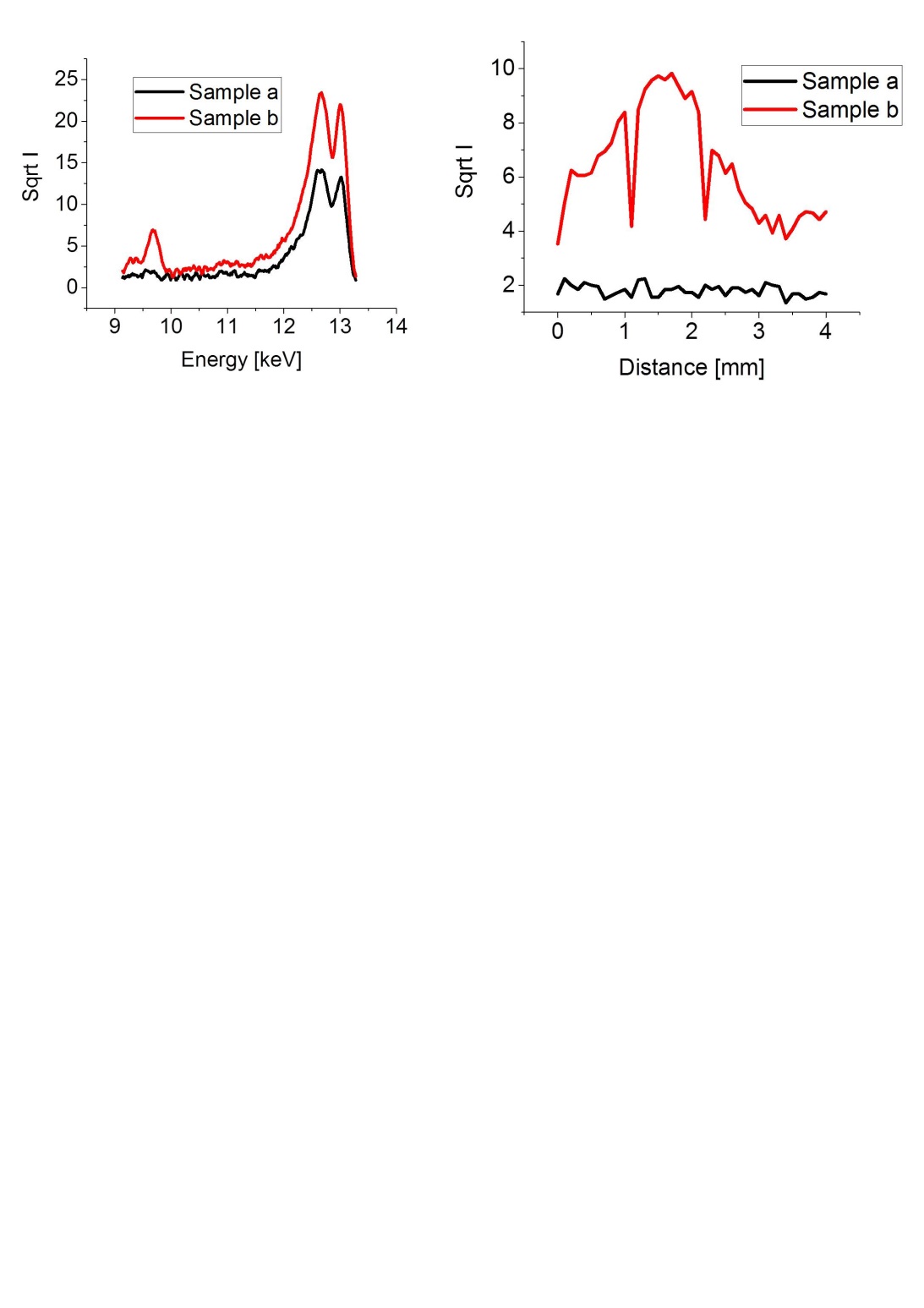


**b**

**a**

Fig S1 **(a)** XRF spectra of the sample from energies for Sample a (black) and Sample b (red). **(b)** XRF spectra of the sample at the L_α_ Au line as function of lateral distance on the Si surface.

L_β_

L_α_

Fig S1 - shows XRF plots for Samples a and b. During the measurements the sample was moved perpendicular to the incident beam with small steps of 0.1 mm. The scanning was made in the central part of the sample, which was equal to 4 mm of the whole 9 mm. Fig S1a shows the XRF yield measured for both samples. The emission lines of Au L_α_ and L_β_ are located at 9.6 keV and 11.4 keV, respectively. They are clearly visible on Sample b, whereas Sample a has no significant signal despite of a larger amount of deposited Au clusters. This can be due to the differences in the sample preparation. Fig S1b gives the XRF yield at an energy of 9.6 keV (corresponding to the L_α_ –line of Au) as function of sample position. This only confirms our supposition that soft-landing deposition scheme is essential for our system.

*Analysis of the spot size from XRF data.*

As an addition to the Fig 2a (of a manuscript) (presented in Fig S2a) in Fig S2b is shown Lorentz fit of the Au spot. This XRF data shows that the cluster spot has a Lorentzian distribution and the average spot size is 0.88 ± 0.02 mm.

**a**

**b**


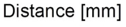

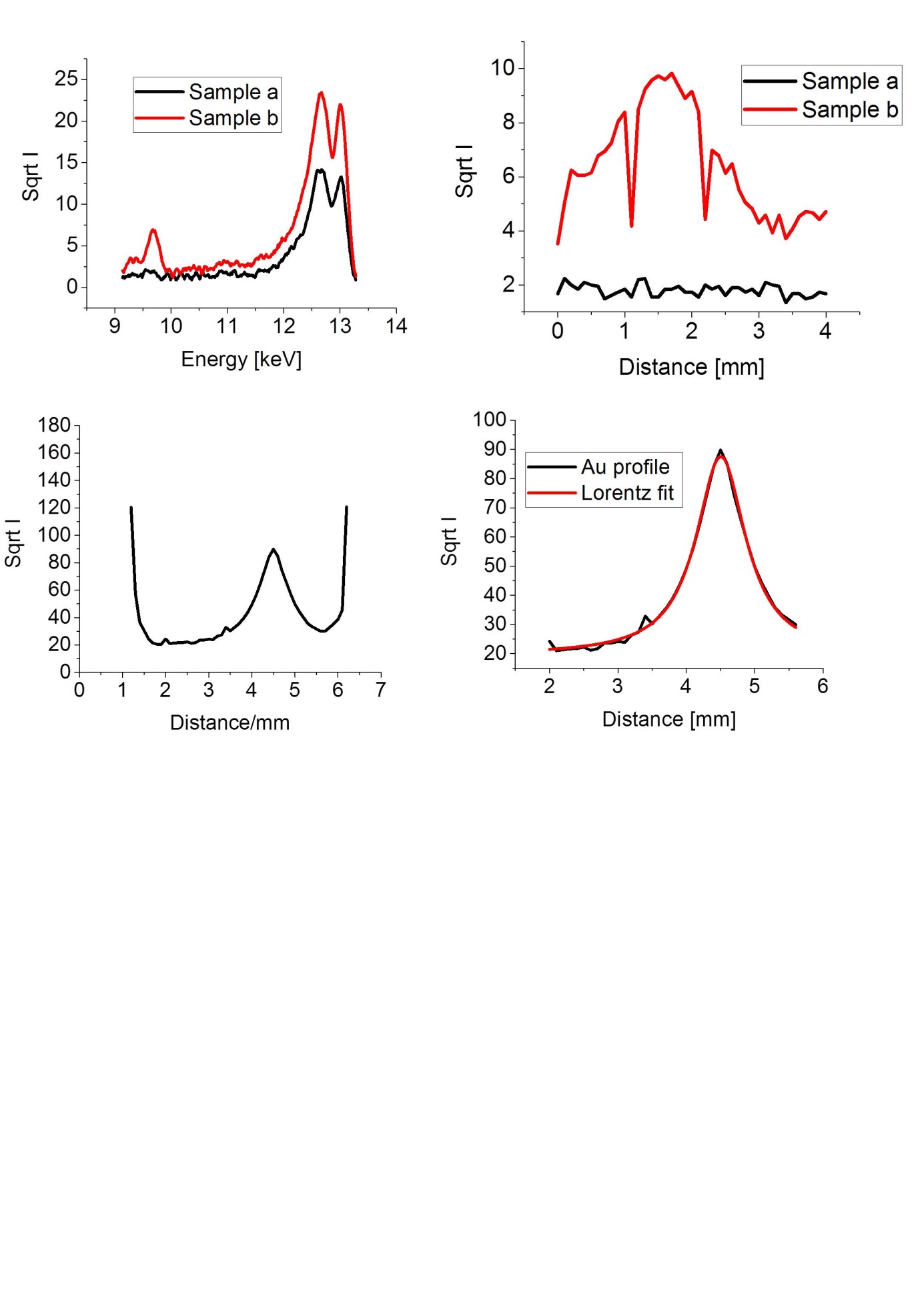


Fig S2 **(a)** XRF spectra of the sample at the L_α_ Au line as function of lateral distance on the Si surface. At a distance of 4.5 mm on x axis is the maximum of XRF signal stemming of the adatomic spot, on the left and right from it are Au markers. **(b)** Part of the XRF spectra with the Lorentz fit.

GISAXS data treatment.

For data analysis DPDAK software package^2^ was used. From the data for the adatomic and Au_9_ samples the background was subtracted. In this case it was the GISAXS plot taken from the scan for the corresponding sample which according to the XRF doesn’t have Au signal. This was done to enhance the signal from the spot. Considering that the whole surface had Al capping layer on top, there shouldn’t be any capping layer contribution in this data.

From the data for the sputtered sample nothing was subtracted due to the fact that the whole surface had Au clusters.

GISAXS out of plane cut analysis.

On the Fig S3a, b, c are shown the fits done by DPDAK analysing plugin. In all the cases Lorentz fit was shown to be the best.

Fig S3a presents the fit for the data in the Fig 2d (manuscript) (adatomic sample), while Fig S3b is the fit for Fig 2f (sputtered sample).

The q_y,max_ in the first case was estimated as -0.961 ± 0.009 nm^-1^, for the second -0.286 ± 0.003 nm^-1^.

In Fig. S3c is shown the fit for Fig.3d where the best fit had q_y,max_ = -2.392 ± 0.149 nm^-1^. This is an expected value for ultrasmall clusters.


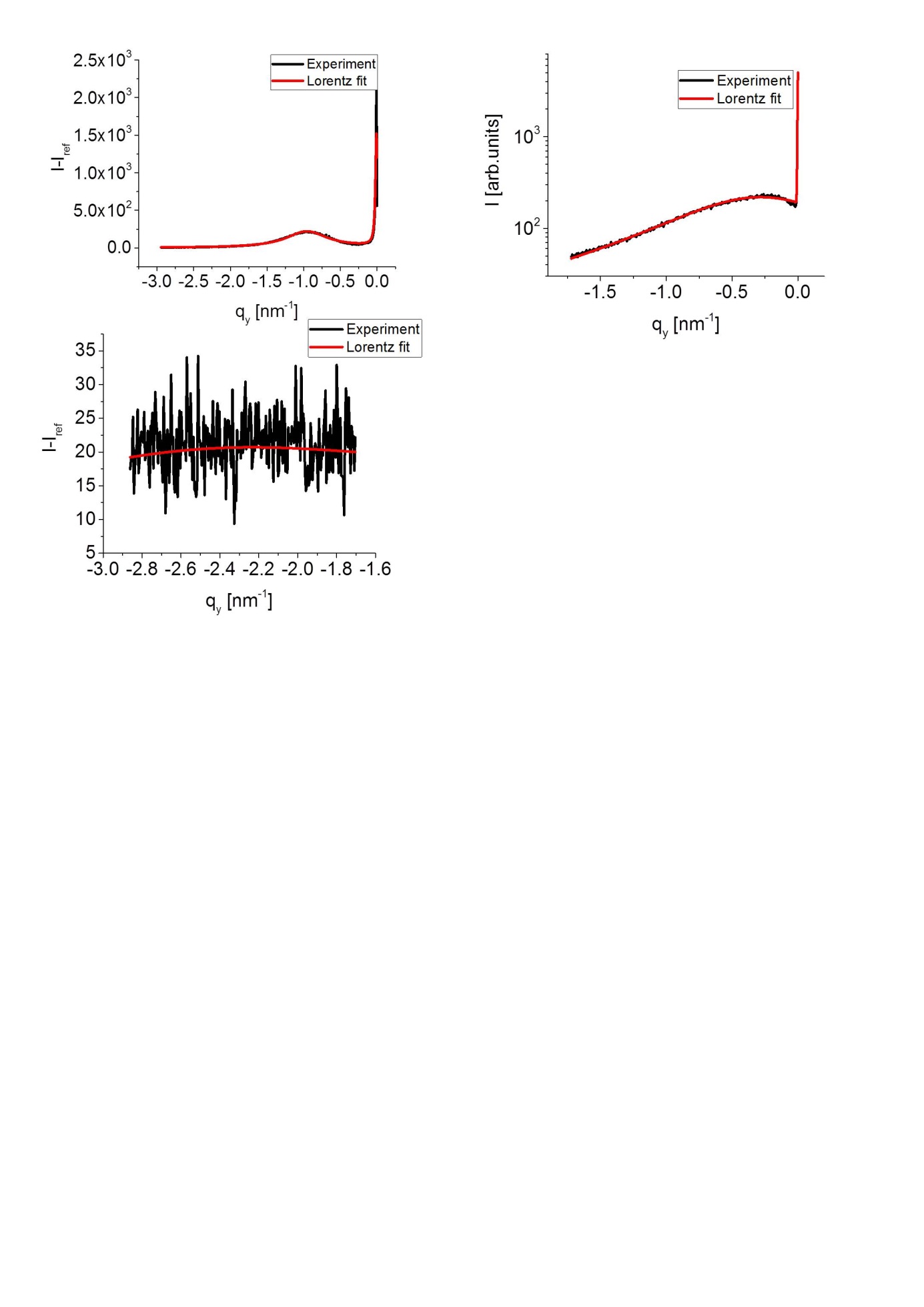


**b**

**c**

**a**

Fig. S3. Out of plane cuts with their fits for samples: **(a)** Adatomic sample, **(b)** Sputtered sample, **(c)** Au_9_ sample.

WAXS data analysis.

For an adatomic and Au_9_ sample GIWAXS measurements were made.

In GIWAXS data analysis, one usually extracts intensity distributions by radial integration to obtain a diffractogram, where peak positions and shape can give the preferred crystallographic lattice according to Bragg´s law and the crystallite size of the object according to Scherrer´s formula^3,4^.


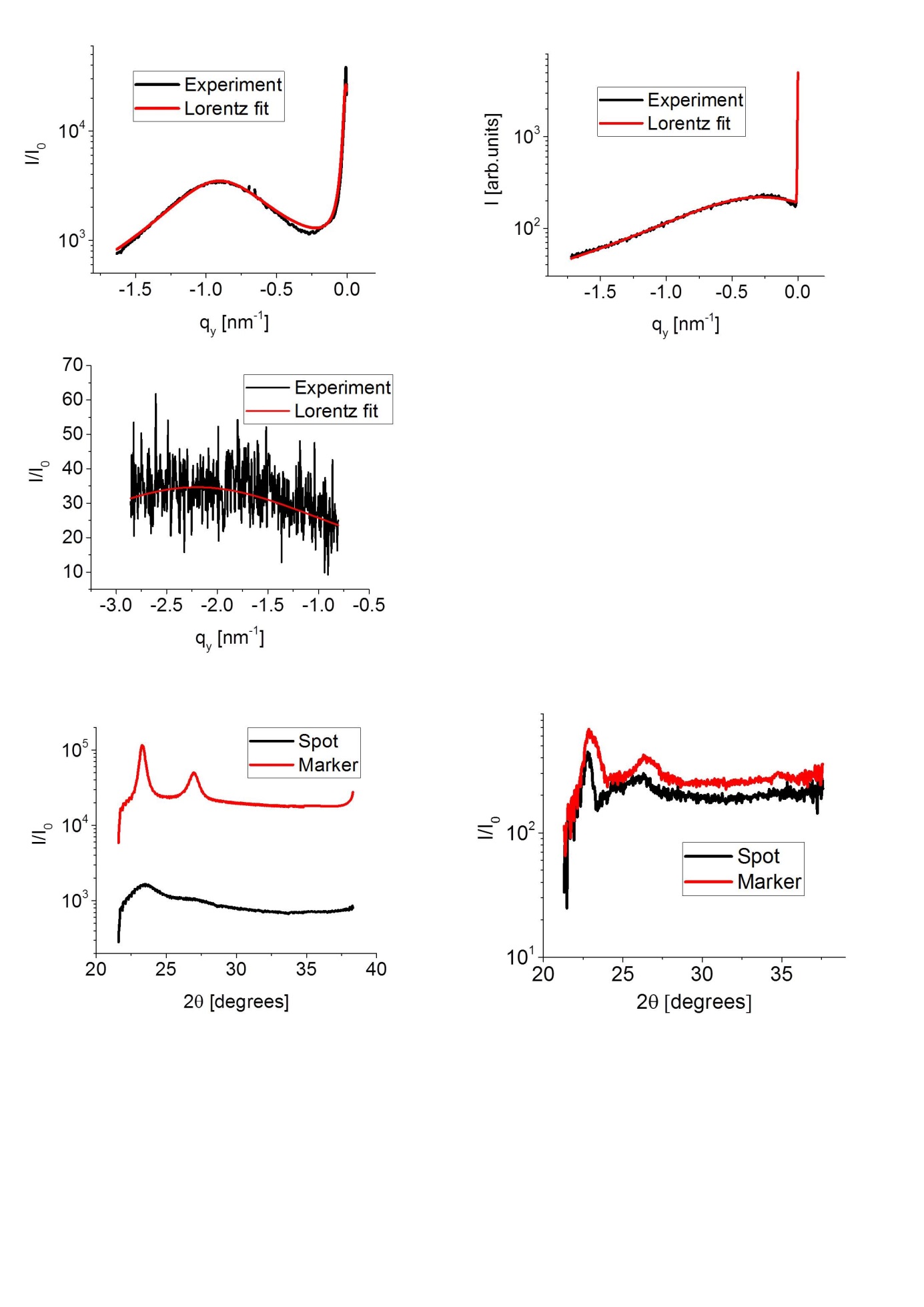
 Fig. S4. GIWAXS diffractograms. **(a)** Diffractogram from the 50%ML Au_1_ sample for the adatomic spot (black) and Au marker (red). **(b)** Diffractogram from the 10%ML Au_9_ sample for the Au_9_ spot (black) and Au marker (red).

**a**

**b**

Au (200)

Au (111)

Au (111)

Au (200)

Fig. S4 is showing diffraction pattern from the spot and marker for both samples. The areas which were used as background for subtraction were chosen according to the corresponding areas on the XRF plots taken simultaneously with the scattering measurements. On all this data background is already subtracted and this explains why there is not Si peak or the one from the capping layer visible. The WAXS pattern for the adatomic sample clearly shows diffraction peaks at positions 2θ = 23.3° and 27.0° for the marker. They correspond to Au (111) and (200) lattice planes. The peak belonging to the adatomic spot is much wider than the one from the marker. This is an expected behaviour of a small structure. In the Au_9_ sample case in Fig S4b we also observe two peaks on the similar positions. A very sharp peak of Au_9_ cluster spot which looks alike the marker one can be explained by a small amount of material with a comparable thickness with the marker. This could happen that on one of the edges perpendicular to the markers the mask wafer shifted and there was a small streak of Au deposited. To quantify the data from GIWAXS measurements particle/crystalline size has been calculated by using Debye-Scherrer formula by^4,5^:

Crystalline size =$\frac{0.9\lambda}{Bcos\theta}$ (4),

where B=$\sqrt{(w_{sample}^{2}-w_{reference}^{2})}$, *w_sample_* is the full width at half maximum (FWHM) of object peak (111) and *w_reference_* is FWHM from the calibrant measured in same experimental conditions (we used LaB_6_). The crystal size for the cluster formed from the adatomic deposition results to 3 nm, the one for Au_9_ is 10 nm and for the marker is about 8.5 nm. This estimation proves that the crystalline size cannot belong to Au_9_ cluster. In addition, if 50% ML gives such a weak signal in comparison to the marker in Fig. S4b we cannot expect any significant peak for only 10% ML.

IsGISAXS simulations.

IsGISAXS^6^ allows to choose the type of a simulated system, which in our case was DWBA_Layer_Island. This simulates that particles are covered with the capping layer on top and they look like islands embedded inside. We used as a substrate Si and Al_2_O_3_ as a capping layer cause Al is easily oxidized being in ambient conditions. The thickness of a capping layer was chosen to be 5 nm because this is the estimated amount according to the Quartz micro balance measurements for the real samples. The wavelength, sample to the detector distance and the detector size was chosen to be 0.0953 nm, 2368 mm and 981 x 1043 (Pilatus 1M), respectively. The shape was AnisoHemiSpheroid, since this could describe the real shape better. The distances such as the one between the clusters and the radius were as calculated from the experiment (Table 2). The height to the radii ratio was varied to fulfil the condition that the cluster has the height of one, two or three Au atoms. This was taken equal to 0.288 nm, which is two covalent radii of Au.

Test for the radiation induced changes.

**b**

**a**


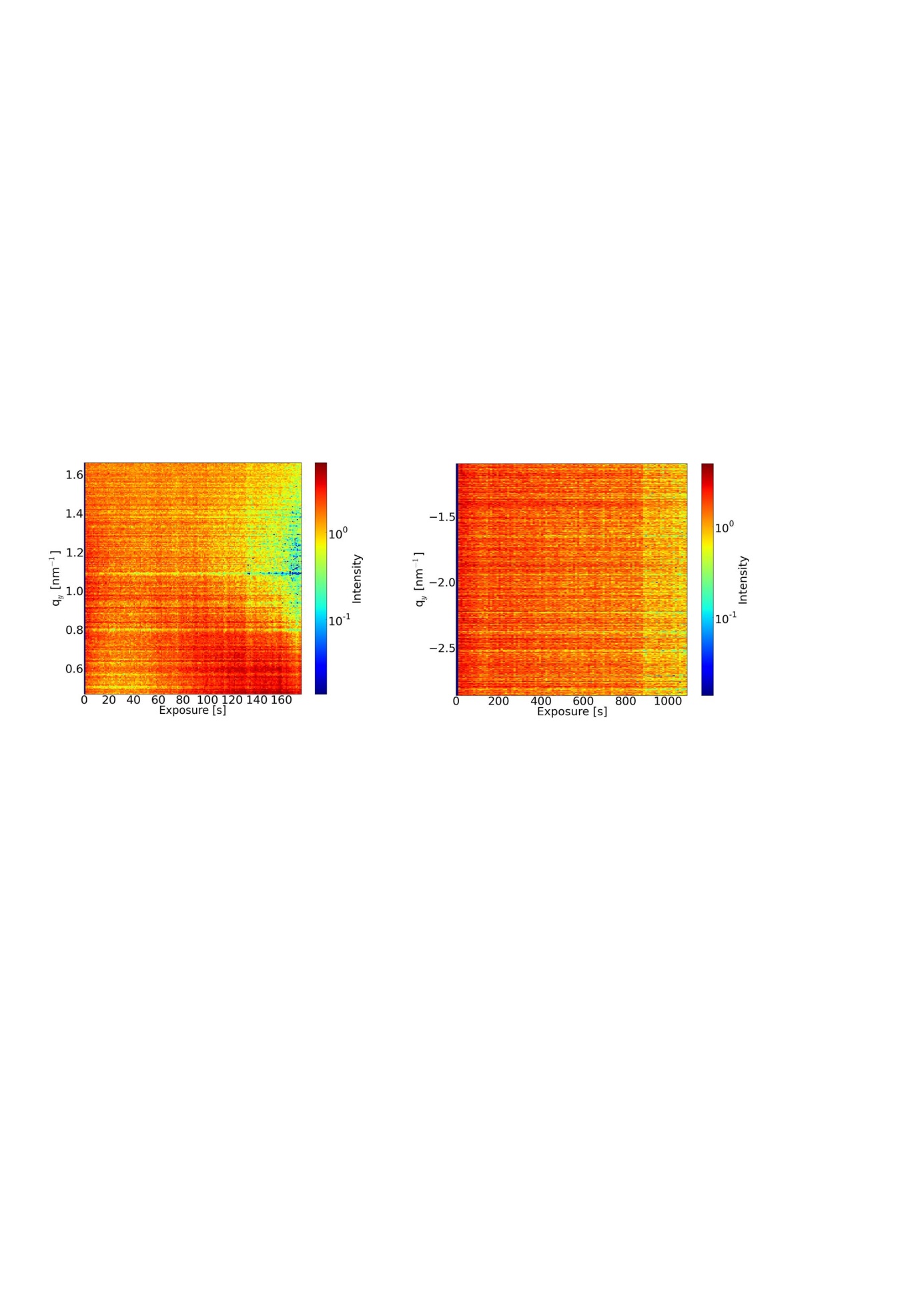


Fig. S5. Map of the out-of-plane cuts (along q_y_) derived at the Si Yoneda peak (q_z_=0.7 nm^-1^) for the q_y,max_ area where is signal for each cluster. The black arrow indicates the frame up to which the data was summed up. **(a)** Adatomic sample with 50%ML**. (b)** Au_9_ sample with 10%ML.

2D maps in Fig. S5. illustrate our selection process for summing up frames which don’t show radiation induced changes. Due to the unstable structures which adatomic sample formed, changes started earlier than for the size-selected clusters caused by their initial shape stability.

Investigation of bare substrate

Si wafer was cleaned according to the usual procedure, described in the Materials and Methods. Data obtained for the bare substrate is in the Fig. S6 and S7. It was investigated using GIWAXS and XPS. In the raw data in Fig. S6 spots from SiO_2_ (Hexagonal lattice, a=b=0.4912 nm, c=0.5404 nm, α=β=90°, γ=120°) are clearly visible, revealing single crystalline structure. Signals on the positions [002] (2θ=20.3146°) and [022] (2θ=33.131°) are pronounced. In the Fig. S7 (calibrated data) Si 2p and SiO2 2p peaks are visible which proves that the deposition was made on the oxide layer of the substrate.


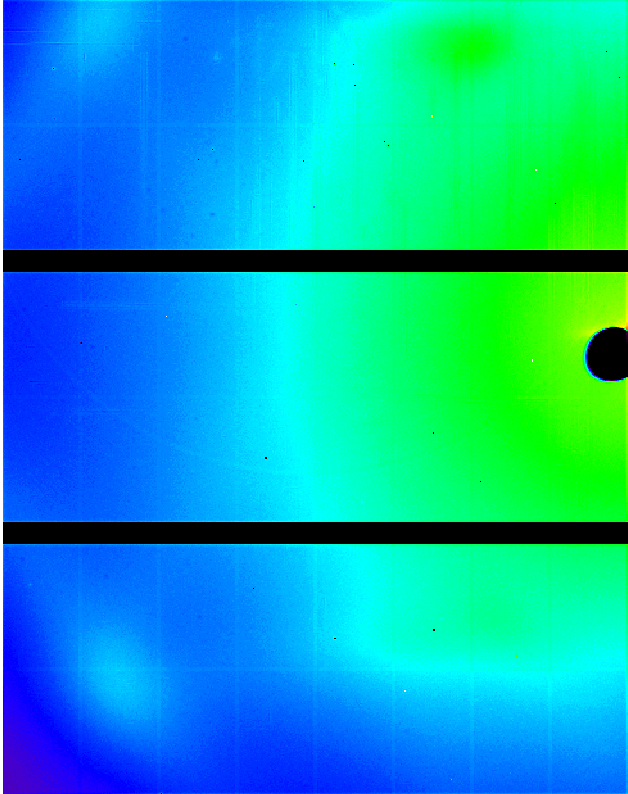


[022]

[002]

[022]

[002]

Fig. S6. GIWAXS detector image of the bare Si/SiO_2_ substrate.

Fig. S7. XPS spectra for bare Si/SiO_2_ substrate of the Si 2p and SiO_2_ 2p peaks.

References

1. Lau, J. T. *et al.* Ultrahigh vacuum cluster deposition source for spectroscopy with synchrotron radiation. *Rev. Sci. Instrum.* **76,** 63902 (2005).

2. Benecke, G. *et al.* A customizable software for fast reduction and analysis of large X-ray scattering data sets: applications of the new DPDAK package to small-angle X-ray scattering and grazing-incidence small-angle X-ray scattering. *J. Appl. Crystallogr.* **47,** 1797–1803 (2014).

3. Begum, P., Bhattacharjee, D., Mishra, B. K. & Deka, R. C. Density functional study on structures, stabilities, and electronic properties of size-selected Pd n Si q (n = 1–7 and q = 0, +1, −1) clusters. *Theor. Chem. Acc.* **133,** 1418 (2013).

4. Langford, J. I. & Wilson, A. J. C. Scherrer after sixty years: A survey and some new results in the determination of crystallite size. *J. Appl. Crystallogr.* **11,** 102–113 (1978).

5. Vinila, V. S., Jacob, R., Mony, A., Nair, H. G. & Issac, S. XRD Studies on Nano Crystalline Ceramic Superconductor PbSrCaCuO at Different Treating Temperatures. *Cryst. Struct. Theory Appl.* **3,** 1–9 (2014).

6. Lazzari, Â. IsGISAXS : a program for grazing-incidence small- angle X-ray scattering analysis of supported islands research papers. *Interfaces (Providence).* 406–421 (2002). doi:10.1107/S0021889802006088
